# Application of network pharmacology in traditional Chinese medicine for the treatment of cardiac diseases 

**DOI:** 10.3389/fphar.2025.1703454

**Published:** 2025-11-14

**Authors:** Xi Zhai, Xingyu Chen, Xiao Xiao, Lan Wu

**Affiliations:** 1 School of Health Science and Engineering, University of Shanghai for Science and Technology, Shanghai, China; 2 Institute of Wound Prevention and Treatment, Shanghai University of Medicine and Health Sciences, Shanghai, China; 3 Shanghai Key Laboratory of Molecular Imaging, Zhoupu Hospital, Shanghai University of Medicine and Health Sciences, Shanghai, China; 4 School of Basic Medical Science, Shanghai University of Medicine and Health Sciences, Shanghai, China; 5 School of Clinical Medical Science, Shanghai University of Medicine and Health Sciences, Shanghai, China

**Keywords:** network pharmacology, traditional Chinese medicine, cardioprotection, cardiac diseases, mechanisms

## Abstract

Many innovative traditional Chinese medicines (TCMs) play a significant role in cardioprotection against cardiac diseases by addressing the basic causes of heart attack, which presents as a dual deficiency of qi and yang. Network pharmacology, offering a multi-dimensional perspective, can elucidate the specific mechanisms of the active components of TCM via a system pharmacology strategy. The methodology of network pharmacology is highly consistent with the modernization of TCM and provides a new perspective and theoretical basis for both basic research and its supplementary clinical research on cardiac diseases. This review summarizes the steps, databases, and software used in network pharmacology systematically. It also discusses the current achievements in applying network pharmacology to understand the mechanisms of some important TCMs (Huangqi, Renshen, and Danshen) and their active components in the context of cardioprotection against cardiac diseases based on a comprehensive literature search on PubMed. Anti-inflammation, anti-oxidation, anti-apoptosis, anti-pyroptosis, and regulation of the PI3K–AKT–mammalian target of the rapamycin (mTOR) signaling pathway were identified as the main mechanisms through which these TCMs exert cardioprotective effects. In addition, this approach provides new ideas for the cure of cancer-induced cardiac injury through network pharmacology.

## Introduction

Cardiac diseases are the leading causes of morbidity and mortality worldwide (Mahmood et al., 2014). The World Health Organization estimates that cardiac diseases account for 17.9 million deaths per year (World Health Organization, 2023). Studies have demonstrated that traditional Chinese medicine (TCM) has potential protective effects in treating various cardiac diseases. Although TCM is criticized or even dismissed by some Western scientists because of its complex composition and unclear therapeutic mechanisms, many randomized controlled trials (RCTs) have spurred its modernization in China (Hao et al., 2017). TCM is now proven to exhibit significant therapeutic efficacy, relatively low toxicity, and favorable cost-effectiveness (Lan et al., 2024; Wang et al., 2024; Wang et al., 2017). However, the modernization of TCM still faces challenges because of its inherent concept of multi-component, multi-target, and multi-pathway properties, especially making the elucidation of its molecular mechanisms challenging. Hence, investigating the molecular mechanisms in basic research plays a crucial role in addressing the challenges in improving TCM modernization, filling the gaps in clinical research.

Network pharmacology (NP) is a comprehensive discipline, integrating systems biology, bioinformatics, pharmacology, and computer science (Friboulet and Thomas, 2005). It establishes a data analysis database, extracts relevant information, and uses relevant software to analyze data to construct a multi-dimensional “drug–target–disease” interaction network (Zhang et al., 2023). Network pharmacology can elucidate the interconnected relationships between syndromes, diseases, and TCM components, focusing on multi-component, multi-channel, and multi-target synergy (Hopkins, 2008). Thereby, it contributes to addressing the challenges in improving TCM modernization from the perspective of basic research and its supplementary clinical research.

Astragali Radix (Huangqi), Ginseng Radix (Renshen), and Salviae Miltiorrhiza Radix (Danshen) are common but key TCMs reported in many treatments for cardiac diseases (Wang et al., 2023). The components of these TCMs are of great significance, and a network pharmacology strategy is required to elucidate the underlying mechanisms. This review summarizes the achievements of applying network pharmacology in these TCMs for treating cardiac diseases based on a comprehensive literature search on PubMed. It aims to create a reference for TCM in treating cardiac diseases through applying network pharmacology and provides new ideas for the cure of cancer-induced cardiac injury through network pharmacology.

## Overview of network pharmacology

### Network pharmacology steps

**FIGURE 1:**
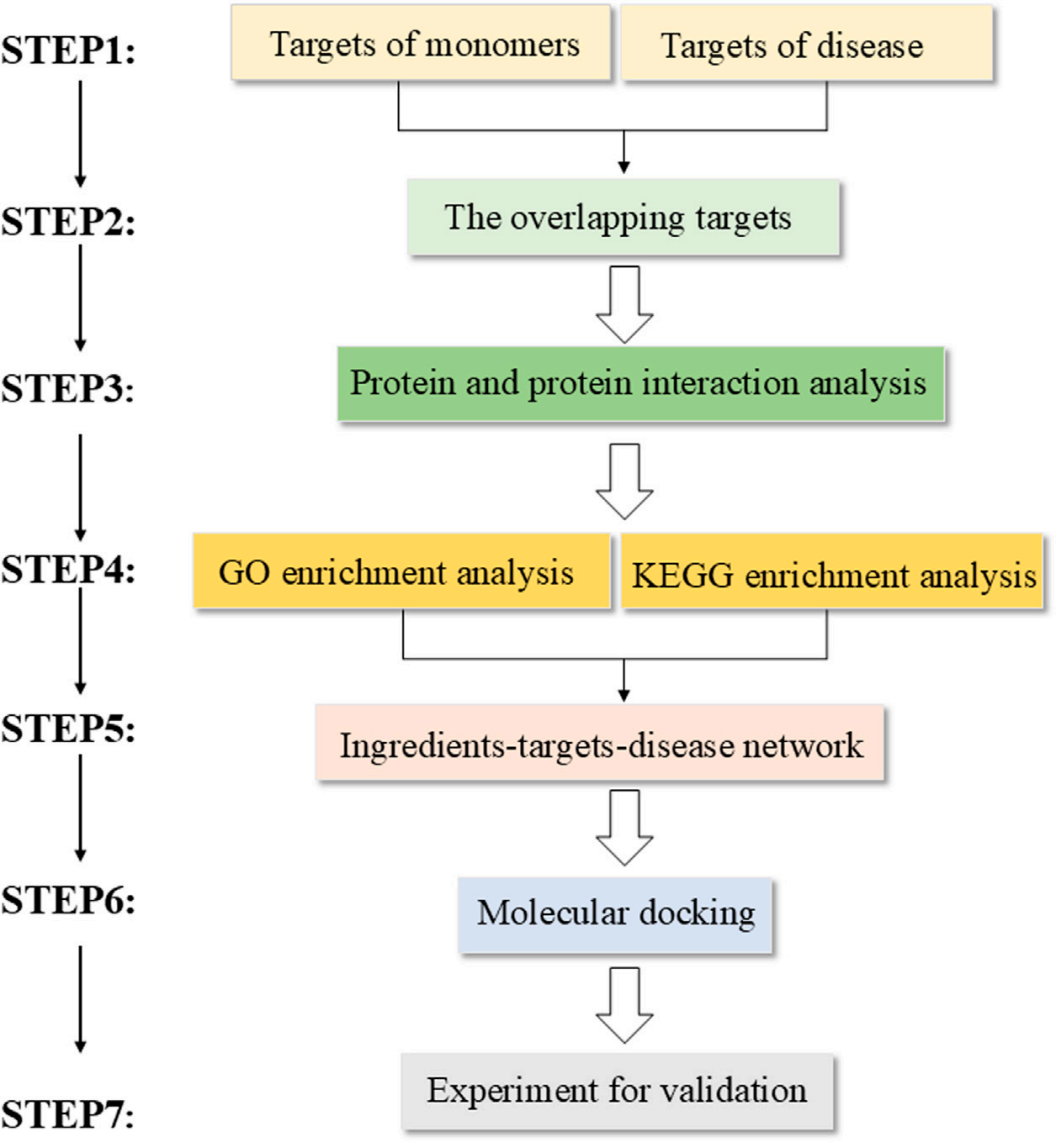
Standardized workflow of network pharmacology.

Network pharmacology follows a standardized workflow (Figure 1). First, the active monomers of drugs are screened, and then their corresponding targets are identified by predicting their potential targets. Second, disease-associated targets are collected from a specialized database. Subsequently, the intersection of drug-predicted targets and disease-associated targets is obtained. Next, the overlapping genes are used to construct a protein–protein interaction (PPI) network to identify key proteins, and the network is used for functional enrichment analyses to understand their biological roles. An “active ingredient–target–pathway” network is mapped utilizing the obtained information. The hub genes and the corresponding drug compounds are screened. Molecular docking is conducted between the screened hub genes and the corresponding high-degree drug monomers identified through topology analysis, and the binding energies are recorded. Finally, validation of experimental results is done through *in vitro* or *in vivo* studies. The websites of the database and tools are listed (Table 1).

**TABLE 1:** Websites of the database and tools in network pharmacology.

Name	Website	Function
DrugBank	https://go.drugbank.com/	TCM databases with reported targets
Traditional Chinese Medicine Systematic Pharmacology (TCMSP)	https://www.tcmsp-e.com/load_intro.php?id=43	TCM databases with reported targets
Traditional Chinese Medicine Integrated Database (TCMID)	http://www.megabionet.org/tcmid/	TCM databases with reported targets
Integrative Pharmacology-based Research Platform of Traditional Chinese Medicine (TCMIP)	http://www.tcmip.cn/	TCM databases with reported targets
Herbal Ingredients' Targets (HIT)	http://www.badd-cao.net:2345/	TCM databases with reported targets
A high-throughput experiment and reference-guided database of TCM (HERB)	http://herb.ac.cn/	TCM databases with reported targets
TCMGeneDIT	—	TCM databases with reported targets
Natural Product Activity and Species Source (NPASS)	http://bidd2.nus.edu.sg/NPASS/	TCM databases with reported targets
ChEMBL	https://www.ebi.ac.uk/chembl/	TCM databases with reported targets
The Encyclopedia of Traditional Chinese Medicine (ETCM)	http://www.tcmip.cn/ETCM/	TCM databases with predicted targets
BATMAN-TCM	https://www.bohrium.com/apps/batmantcm	TCM databases with predicted targets
Swiss Target Prediction	https://swisstargetprediction.ch/	TCM databases with predicted targets
Pharm Mapper	https://www.lilab-ecust.cn/pharmmapper/index.html	TCM databases with predicted targets
SuperPred	https://prediction.charite.de/	TCM databases with predicted targets
Similarity Ensemble Approach (SEA)	https://sea.bkslab.org/	TCM databases with predicted targets
DisGeNET	http://www.disgenet.org/	Databases for disease targets
Gene Expression Omnibus (GEO)	https://www.ncbi.nlm.nih.gov/geo/	Databases for disease targets
MalaCards	https://www.malacards.org/	Databases for disease targets
The Human Phenotype Ontology (HPO)	https://hpo.jax.org/	Databases for disease targets
The Pharmacogenomics Knowledge Base (PharmGKB)	https://www.pharmgkb.org/about	Databases for disease targets
Therapeutic Target Database (TTD)	https://db.idrblab.net/ttd/	Databases for disease targets
GeneCards	https://www.genecards.org/	Databases for disease targets
Online Mendelian Inheritance in Man (OMIM)	https://www.omim.org/	Databases for disease targets
Venny	https://bioinfogp.cnb.csic.es/tools/venny/	Drug–disease common targets
DAVID	https://davidbioinformatics.nih.gov/	Gene Ontology and Kyoto Encyclopedia of Genes and Genomes enrichment analyses
Metascape	http://www.bioinformatics.com.cn/	Gene Ontology and Kyoto Encyclopedia of Genes and Genomes enrichment analyses
STRING	https://cn.string-db.org/	Protein–protein interaction analysis
Cytoscape	—	Protein–protein interaction analysis
PubChem	https://pubchem.ncbi.nlm.nih.gov/	Chemical data of database
Protein Data Bank (PDB)	https://www.rcsb.org/	Protein structures of database
AutoDock Vina	http://vina.scripps.edu/	Molecular docking
Discovery Studio (DS)	—	Molecular docking
Schrödinger	—	Molecular docking

### Databases for TCM

Some important databases that provide information on monomers and their reported targets include DrugBank, Traditional Chinese Medicine Systematic Pharmacology (TCMSP) (Liu et al., 2022), Traditional Chinese Medicine Integrated Database (TCMID), Integrative Pharmacology-based Research Platform of Traditional Chinese Medicine (TCMIP), Herbal Ingredients’ Targets (HIT), a high-throughput experiment- and reference-guided database of TCM (HERB), TCMGeneDIT, ChEMBL, Traditional Chinese Medicine on Immuno-Oncology (TCMIO), and Integrated Traditional Chinese Medicine (iCTM). Some databases focus on the interactions between drugs, their components, and their predicted targets such as Bioinformatics Analysis Tool for Molecular Mechanism of Traditional Chinese Medicine (BATMAN-TCM), Encyclopedia of Traditional Chinese Medicine (ETCM), PharmMapper, SuperPred, SwissTargetPrediction, Similarity Ensemble Approach (SEA), TCM Database@Taiwan, and Natural Product Activity and Species Source (NPASS) database (Gong et al., 2023).

### TCM databases with reported targets

TCMSP is a unique system pharmacology platform of Chinese herbal medicines (Wu, et al., 2022) that offers information about the components of Chinese herbal medicines from the Chinese Pharmacopoeia, disease details targeted by each active component, and absorption, distribution, metabolism, and excretion (ADEM) properties of drugs, such as oral bio-availability (OB), drug-likeness (DL), and half-life (HL). The screening criteria are usually OB ≥ 30% and DL ≥ 0.18, which ensures the pharmacological validity and bio-safety profile of the selected components. OB is a novel chemo-metric method for the prediction of human oral bio-availability, which represents the amount of the component that enters the systemic circulation. DL is the similarity between a known monomer and a certified drug structure. HL estimates the component metabolic stability. Additionally, it provides the two-dimensional (2D) structures of the components in mol2 file format and visualization of the herb–component–target–disease network (Ru et al., 2014). The TCMSP database primarily integrates information on TCM formulas, herbs, compounds, and targets. However, it is not suitable for Western drugs because of the relatively slow speed of database updates. It is mainly applicable for studying the mechanisms of TCM formulas, TCM monomers, or herbal compounds (Ru et al., 2014). DrugBank is a comprehensive, freely available web resource supplying detailed drug information, drug target, drug interaction, and drug action information (Wishart, 2008). These targets have been experimentally validated and are continuously updated. In addition, the TCMSP platform can be directly linked to DrugBank. The TCMID, TCMIP, HERB, TCMGeneDIT, ChEMBL, HIT, and NPASS databases also provide reported targets of the drugs (Zeng et al., 2018).

### TCM databases with predicted targets

BATMAN-TCM is the first online bioinformatics analysis database specifically designed for studying molecular mechanisms of TCM (Liu et al., 2016). It enables the investigation of holistic mechanisms of TCM formulas/herbs. The platform can predict potential targets through setting a cutoff score and adjusted p-value. A cutoff score is a confidence threshold for filtering target prediction results. An adjusted p-value is a statistical adjustment tool that can reduce the risk of false positives. It can also conduct a comprehensive analysis of enriched biological pathways (BP) and Gene Ontology (GO) functional terms for the targets. It visualizes “drug–target–pathway/disease” interaction networks and supports comparative analysis of multiple TCM formulas.

SwissTargetPrediction is a web-based platform designed to accurately predict potential targets for small molecules by analyzing the 2D and three-dimensional (3D) molecular structural similarity (Gfeller et al., 2014). Users can input molecules through SMILES or the Marvin JS molecular editor to obtain the corresponding targets. The SwissTargetPrediction platform integrates ligand- and target-based similarity methods, resulting in high accuracy for target prediction. However, its functionality is singular, as it only performs target prediction and does not include subsequent network construction and analysis functions. Therefore, its predictions require experimental validation (Daina et al., 2019).

PharmMapper and SuperPred also provide the prediction targets of the drugs. PharmMapper identifies potential drug targets for small-molecule compounds (e.g., drugs and natural products) through pharmacophore mapping. Users can upload a molecular 3D structure file in mol2 format to the PharmMapper server. Optional parameters may be configured, or default settings can be used. Results are accessed by entering tracking JOB ID on the output page (Liu et al., 2010).

SuperPred has three input methods: First, enter the PubChem compound name of the target structure; second, enter/upload the SMILES number of the molecule; third, use the built-in drawing tools to draw or edit the molecular structure, and start the calculation by clicking the button; the output results can be obtained as anatomical therapeutic chemical (ATC) code prediction and target prediction. The ATC code prediction is classified according to the color. Green represents high confidence, yellow represents medium confidence, and red represents low confidence. Target prediction is reported in the form of a table, which provides the target name, ChEMBL ID, UniProt ID, Protein Data Bank (PDB) visualization link, TTDID, binding probability, and model accuracy (Gallo et al., 2022).

SEA predicts drug targets by submitting the compound’s SMILES notation to a search server (Liu Z. et al., 2023).

### Databases for disease targets

The disease-associated targets are derived from databases such as Gene Expression Omnibus (GEO), DisGeNET, Pharmacogenomics Knowledge Base (pharmGKB), Therapeutic Target Database (TTD), GeneCards, Online Mendelian Inheritance in Man (OMIM), Human Phenotype Ontology (HPO), and MalaCards database (Zhao M. et al., 2024). GEO distributes micro-array, next-generation sequencing (NGS), and other forms of high-throughput functional genomics data (Barrett et al., 2013). DisGeNET is a knowledge management platform that covers the full spectrum of human diseases and normal and abnormal traits (Piñero et al., 2020). The DisGeNET platform integrates multiple public databases. However, the reliability of these data varies by source, requiring users to exercise discretion when interpreting them (Piñero et al., 2020). The PharmGKB website provides a diverse array of pharmacogenomics (PGx) information (Barbarino et al., 2018). TTD provides comprehensive information to evaluate durability characteristics of the targets (Zhou et al., 2024). GeneCards is a comprehensive and authoritative compendium that provides annotative information on human genes, containing concise genome, transcriptome, proteome, and function data of all known and predicted human genes (Safran et al., 2010). OMIM is the primary repository that provides comprehensive and curated information on genes, genetic phenotypes, and the relationships between them. Its structure is organized into distinct gene and phenotype entries, which provides external links to target-related information. Each gene is assigned a unique six-digit OMIM identifier for classification. Gene entries include protein-coding genes, regulatory elements, and non-coding RNAs, whereas phenotype entries focus on single-gene-mediated disorders. The database facilitates genomics coordinate searches through their gene maps with precise localization of genes and phenotypes within the human genome (Amberger et al., 2015). The UniProt database is used to standardize all the collected protein names by converting them into their corresponding official abbreviations (Shang et al., 2023).

### Platform for the intersection of disease targets and drug targets

Using the Venny 2.1.0 online platform, overlapping genes can be identified to generate a Venn diagram of disease genes and drug targets. These intersecting genes represent the potential therapeutic targets for drug treatment of the disease (He et al., 2023).

### Platform for Gene Ontology and Kyoto Encyclopedia of Genes and Genomes enrichment analyses

The DAVID website or Metascape database can be used to perform Gene Ontology (GO) and Kyoto Encyclopedia of Genes and Genomes (KEGG) enrichment analyses on potential therapeutic targets screened by TCM and disease databases (Shang et al., 2023). GO enrichment analysis covers biological process (BP), molecular function (MF), and cell composition (CC). GO enrichment analysis enables researchers to understand biological functions, pathways, and locations of gene enrichment in the cells. KEGG enrichment predicts the signaling pathways of diseases cured by drugs. The visualization program of the bioinformatics mapping website creates bubble maps and bar charts. In the chart, fold enrichment represents the ratio of the enrichment level of a target event observed in an experimental group relative to the control group. The p-value is a statistical measure used to evaluate the probability that the observed data align with the null hypothesis.

### Tools for protein–protein interaction analysis

The STRING platform is a comprehensive online resource that systematically collects and integrates PPI data. It employs confidence scores to quantitatively assess interactions, in which higher scores indicate a greater likelihood of functional association between proteins. It can perform cluster analysis among proteins. The clustering algorithms used in STRING include Monte Carlo localization (MCL) and K-means (Szklarczyk et al., 2021). The MCL algorithm exhibits stronger robustness to noise, making it particularly suitable for filtering false positives in PPI networks. The K-means algorithm is more suitable for identifying gene modules with similar expression patterns in PPI networks (Ren et al., 2024). The STRING database integrates PPI data from experimental, database, literature, and computational predictions; however, it primarily focuses on protein interactions and does not directly handle compounds (Zhao and Sahni, 2019).

Cytoscape is a software platform for visualizing complex biological and social networks (Otasek et al., 2019). It covers many plugins such as NetworkAnalyzer, CytoNCA, MCODE, and CytoHubba, which are used for network analysis. NetworkAnalyzer is the preliminary screening tool for understanding the basic characteristics of a newly constructed network (such as a PPI network and co-expression network), verifying whether the network conforms to common topological characteristics of biological networks (such as scale-free), and quickly calculating the number of node connections. The important parameters calculated by NetworkAnalyzer include betweenness centrality (BC), closeness centrality (CC), and degree centrality (DC). BC represents the number of shortest paths passing through a node. CC represents the average distance between the node and other nodes in the network. DC is used to calculate the edges linked to each node, which indicates the significance of the nodes in the network. The first screening usually selects genes with BC, CC, and DC values greater than the mean or selects genes based on some specific standard (Arjmand et al., 2022).

Then, the deep screening for hub genes uses CytoNCA, MCODE, or CytoHubba. CytoNCA also calculates BC, CC, and DC values that deeply explore the importance, influence, and control of nodes in the network, particularly identifying core nodes in the network. The selection for hub genes is based on BC, CC, and DC values given by CytoNCA that are greater than the median (Fan et al., 2023); MCODE performs a cluster analysis for hub genes and identifies potential protein complexes or functional modules by analyzing the density of nodes and edges in the network. The selection for hub genes is based on the scores given by MCODE for the genes (Shang et al., 2023); CytoHubba performs network topology analysis and node centrality analysis through assigning values to each gene and ranking them based on the attributes of nodes in the network. The important topology analysis method in CytoHubba includes maximum clique centrality (MCC), maximum neighborhood component centrality (MNC), edge percolated component (EPC), and degree. MCC identifies the nodes with centrality in the largest group. MNC evaluates the centrality of a node in its neighboring components. EPC finds key nodes in the network that are still connected after removing a small number of edges. Degree calculates the degree of a node, which is the number of edges directly connected to the node. The hub genes are usually selected based on the intersection of the top screening genes through four topology analyses (Bisht et al., 2024). In addition, each version of Cytoscape introduces new plugins and improved features (Saito et al., 2012). The Cytoscape platform has numerous plugins (such as CytoHubba and MCODE) for pathway enrichment and network topology analysis, and it can produce high-quality network diagrams. However, it does not provide its own data. The users must import data from other databases to construct networks (Shannon et al., 2003).

### Tools for molecular docking

PubChem is a key chemical information resource, which offers chemical informatics, chemical biology, medicinal chemistry, and drug discovery for biomedical research communities (Kim et al., 2019). PubChem allows users to download 2D and 3D structures and obtain the SMILES of bioactive compounds.

PDB is the single worldwide archive of structural data of biological macromolecules (Berman et al., 2000). The crystal structures of core protein targets can be downloaded from the PDB. The angstrom (Å) refers to the resolution of proteins, which is a critical parameter for evaluating structural precision. Lower values indicate higher structural precision. Protein structures are primarily determined through three experimental methods: X-ray crystal structure determination, NMR, and 3D electron microscopy (3DEM) (Burley et al., 2017). X-ray crystal structure determination involves irradiating protein crystals with X-rays and analyzing the diffraction patterns to infer atomic positions. NMR determines inter-atomic distances and angles by analyzing the nuclear magnetic resonance signals of proteins in a solution. 3DEM rapidly freezes protein samples and reconstructs 3D structures by capturing multi-angle images via electron microscopy.

AutoDock Vina is a popular program for molecular docking and virtual screening (Trott and Olson, 2010). The binding energy result is visualized in PyMOL and Python. A value less than −5 kcal/mol indicates good docking. AutoDockTools is used to dehydrate and hydrogenate the proteins. Discovery Studio (DS) supports both protein pre-processing and direct execution of molecular docking. Schrödinger can also perform molecular docking directly (Liu X. et al., 2023).

In summary, each platform has its own focus, advantages, and disadvantages. In practical applications, utilizing multiple platforms is the most common approach.

## Key TCM against cardiac diseases by network pharmacology

All the herbs, active compounds, hub genes, and pathways for TCM against different cardiac diseases by NP are listed (Table 2).

**TABLE 2:** Summary of the data from network pharmacology in different cardiac diseases.

Herb/formula	Active compound	Hub gene	Pathways	Evidence type
—	Salvianolic acid A	*SRC*, *CTNNB1*, *PIK3CA*, *AKT1*, *RELA*, *EGFR*, *FYN*, *ITGB1*, *MAPK8*, and *NFKB1*	Lipid and atherosclerosis, Fluid shear stress and atherosclerosis, Focal adhesion, AGE–RAGE signaling pathway in diabetic complications, PI3K–AKT signaling pathway, sphingolipid signaling pathway, HIF-1 signaling pathway, TNF signaling pathway, and IL-17 signaling pathway	Myocardial infarction
*Salvia miltiorrhiza* Bunge	Luteolin, tanshinone IIA, and 1,2,5,6-tetrahydrotanshinone	*IL-6*, *TNF*, *AKT1*, and *VEGFA*	Blood vessel endothelial cell, migration, membrane raft, and cytokine receptor binding	Myocardial infarction
Danshen Yin	Salvianonol, dihydroisotanshinone II, cryptotanshinone, tanshinone IIB, caffeic acid, rosmarinic acid, salvianolic acid A, dihydrotanshinone I, danshenxinkun A, and lithospermic acid	*ERK*, *JNK*, *AKT*, and *SMAD3*	Extracellular matrix organization, focal adhesion, ECM–receptor interaction, and TGF-β-mediated signaling pathways	Myocardial infarction
Ginseng	Ginsenoside Rh2	*IL1B*, *CASP3*, *RELA*, *CASP8*, *MAPK14*, *CASP1*, and *GSTP1*	Lipid and atherosclerosis, TNF signaling pathway, AGE–RAGE signaling, and NOD-like receptor signaling pathway	Myocardial infarction
QishenYiqi dripping pill	Quercetin, isotanshinone Ⅰ, terfenadine, luteolin, and salvilenone	*AKT1*, *BCL-2*, *CASP1*, *GSK-3B*, and *P53*	PI3K–AKT signaling pathway, autophagy in animals, mitophagy in animals, mTOR signaling pathway, p53 signaling pathway, and NOD-like receptor signaling pathway	Myocardial ischemia–reperfusion injury
Cinnamon	Oleic acid, palmitic acid, β-sitosterol, eugenol, taxifolin, and cinnamaldehyde	*PTGS2*, *GSK-3B*, and *MAPK14*	Lipid and atherosclerosis, PI3K–AKT signaling pathway, MAPK signaling pathway, IL-17 signaling pathway, and HIF-1 signaling pathway	Myocardial ischemia–reperfusion injury
Shuxin decoction	Quercetin, β-sitosterol, and kaempferol	*IL-6*, *IL1B*, *TNF*, *VEGFA*, *MMP9*, *CXCL8*, *STAT3*, *PTGS2*, *CASP3*, *JUN*, *PPARG*, *HIF-1A*, *IL-10*, *ICAM1*, *NOS3*, *HMOX1*, *FOS*, *MYC*, *IFNG*, *EDN1*, *CASP8*, *MAPK8*, *VCAM1*, *CCND1*, *SERPINE1*, *MAPK14*, *STAT1*, *ESR1*, *MPO*, *NOS2*, *CASP1*, *SPP1*, *IL1A*, *SELE*, *NFE2L2*, *CASP9*, *PPARA*, *KDR*, *CXCL10*, and *CD40LG*	AGE–RAGE signaling pathway in diabetic complications, lipids and atherosclerosis signaling pathway, apoptosis, LDL, AGEs, and RAGE	Myocardial ischemia–reperfusion injury
—	Salvianolic acid B	*ALB*, *CASP3*, *ANXA5*, *NOS1*, *SRC*, *NOS3*, *MAPK14*, *MAPK8*, *MAPK1*, and *PPARG*	Cell death-related signaling pathway, inflammation reaction-related signaling pathway, and oxidative stress reaction-related signaling pathways	Myocardial ischemia–reperfusion injury
*Panax ginseng* C.A. Mey	Ginsenoside Rh4, ginsenoside Rk3, ginsenoside Rk1, ginsenoside Rg5, and ginsenoside CK	*EGFR*, *AKT1*, *ERBB2*, *STAT3*, *TNF*, *ESR1*, *mTOR*, *HRAS*, *MMP9*, and *PIK3CA*	PI3K–AKT signaling, TNF signaling pathway, and mTOR signaling pathway	Heart failure
*Astragalus membranaceus*	Isorhamnetin, quercetin, calycosin, formononetin, and kaempferol	*APOE*, *TNF*, *BCL-2*, *MYC*, *MMP9*, *TLR4*, *ESR1*, *HIF-1A*, *VCAM1*, and *CDH1*	Organic hydroxyl compounds and other metabolic processes, cell apoptosis, adhesion ability, and inflammatory response (upregulated); secondary metabolism, the regulation of vascular diameter, steroid hormone response, and cell growth and senescence (downregulated)	Heart failure
Ginseng	Ginsenoside Rg1 and ginsenoside Rb3	*FN1* and *PRKAA2*	Hypertrophic cardiomyopathy, starch and sucrose metabolism, tyrosine metabolism, PI3K–AKT signaling pathway, and AMPK signaling pathway	Heart failure
Tonifying kidney and activating blood	Quercetin, luteolin, kaempferol, tanshinone IIA, and baicalein	*TNF*, *AKT1*, *STAT3*, RELA, *NFKBIA*, and MAPK14	Lipid and atherosclerosis, TNF signaling, PI3K–AKT signaling, and pathways in cancer	Heart failure
Ginseng	Kaempferol, β-sitosterol, and fumarine	*CCNA2*, *STAT1*, and *ICAM1*	AGE–RAGE signaling pathway in diabetic complications, fluid shear stress and atherosclerosis pathway, and TNF signaling pathway	Drug-induced cardiotoxicity
*Salvia miltiorrhiza*	Danshensu	*CAT*, *SOD*, *GPX*, *IL-6*, *TNF*, *BAX*, *BCL-2*, and *CASP3*	Oxidative stress, apoptosis, inflammation, heart development, negative regulation of cell growth, cell aging, negative regulation of autophagy, ubiquitin protein ligase binding, NF-κB binding, antioxidant activity, and tumor necrosis factor-activated receptor activity	Drug-induced cardiotoxicity
Danshen injection	Chrysophanol, luteolin, tanshinone IIA, and isoimperatorin	*CA12*, *NOS3*, and *POLH*	Neuroactive ligand–receptor interaction, apoptosis, and nitrogen metabolism and calcium signaling pathways	Drug-induced cardiotoxicity
Huangqi–Danshen compound	Salvianolic acid B, rosmarinic acid, astragaloside IV, tanshinones, and tanshinins	*SOD*, *CASP3*, *BCL-2*, *EDN1*, *NRF2*, *AMPK*, *mTOR*, and *PGC1A*	FoxO signaling pathway, insulin signaling pathway, Ras signaling pathway, HIF-1 signaling pathway, estrogen signaling pathway, insulin resistance, PPAR signaling pathway, VEGF signaling pathway, PI3K–AKT signaling pathway, ErbB signaling pathway, complement and coagulation cascades, adipocytokine signaling pathway, metabolic pathways, AMPK signaling pathway, TNF signaling pathway, mTOR signaling pathway, glucagon signaling pathway, MAPK signaling pathway, and the Toll-like receptor signaling pathway	Diabetic cardiomyopathy

Cadherin-associated protein b 1(*CTNNB1*), phosphatidylinositol 3-kinase catalytic subunit alpha (*PIK3CA*), protein kinase B (*AKT1*), recombinant V-Rel reticuloendotheliosis viral oncogene homolog A (*RELA*), epidermal growth factor receptor (*EGFR*), integrin subunit beta 1 (*ITGB1*), mitogen-activated protein kinase 8 (*MAPK8*), nuclear factor κβ subunit 1 (*NFKB1*), interleukin-6 (*IL-6*), tumor necrosis factor (*TNF*), vascular endothelial growth factor A (*VEGFA*), B-cell lymphoma-2 (*BCL-*2), glycogen synthase kinase-3β (*GSK-3B*), hypoxia-inducible factor 1 (*HIF-1*), prostaglandin-endoperoxide synthase 2 (*PTGS2*), C–X–C motif chemokine ligand 8 (*CXCL8*), caspase-3 (*CASP3*), tumor protein P53 (*P53*), Jun proto-oncogene, AP-1 transcription factor subunit (*JUN*), interleukin-10 (*IL-10*), nitric oxide synthase 3 (*NOS3*), heme oxygenase 1 (*HMOX1*), Fos proto-oncogene, AP-1 transcription factor subunit (*FOS*), MYC proto-oncogene, BHLH transcription factor (*MYC*), interferon gamma (*IFNG*), endothelin 1 (*EDN1*), serpin family E member 1 (*SERPINE1*), mitogen-activated protein kinase 14 (*MAPK14*), signal transducer and activator of transcription 1 (*STAT1*), myeloperoxidase (*MPO*), nitric oxide synthase 2 (*NOS2*), caspase-1 (*CASP1*), secreted phosphoprotein 1 (*SPP1*), interleukin-1α (*IL1A*), selectin E (*SELE*), nuclear factor, erythroid 2-like 2 (*NFE2L2*), caspase 9 (*CASP9*), peroxisome proliferator-activated receptor α (*PPARA*), kinase insert domain receptor (*KDR*), C–X–C motif chemokine ligand 10 (*CXCL10*), CD40 ligand (*CD40LG*), albumin (*ALB*), annexin A5 (*ANXA5*), epidermal growth factor receptor-binding protein 2 (ERBB2), signal transducer and activator of transcription 3 (*STAT3*), estrogen receptor a1 (*ESR1*), Harvey rat sarcoma viral oncogene homolog (*HRAS*), matrix metalloproteinase-9 (*MMP9*), mammalian target of rapamycin (*mTOR*), apolipoprotein E (*APOE*), Toll-like receptor 4 (*TLR4*), vascular cell adhesion molecule 1 (VCAM1), cadherin-1 (*CDH1*), nuclear factor κβ subunit inhibitor α (NFKBIA), fibronectin 1 (*FN1*), protein kinase AMP-activated catalytic subunit alpha 2 (*PRKAA2*), adenosine 5′-monophosphate (AMP)-activated protein kinase (*AMPK*), cyclin A2 (*CCNA2*), signal transducer and activator of transcription 1 (*STAT1*), intercellular adhesion molecule 1 (*ICAM1*), catalase (*CAT*), superoxide dismutase (*SOD*), glutathione peroxidase (*GPX*), carbonic anhydrase 12 (*CA12*), DNA polymerase eta (*POLH*), advanced glycation end products (AGE), the receptor for AGEs (RAGE), nuclear factor-E2-related factor 2 (*NRF2*), peroxisome proliferator-activated receptor γ (*PPARG*), PPARγ coactivator 1α (*PGC1A*).

### Myocardial infarction

Myocardial infarction (MI) represents myocardial necrosis due to persistent ischemia and hypoxia due to coronary artery occlusion. SwissTargetPrediction, HERB, TargetNet, GeneCards, OMIM, DisGeNET, and TTD were used to predict the potential targets of salvianolic acid A. PPI networks were constructed using the STRING database, and the CytoHubba plugin within Cytoscape was utilized to identify the hub genes. Functional enrichment analysis of these hub genes was performed using the clusterProfiler software package. Experiments confirmed that salvianolic acid A regulates the expression of these hub genes, thereby demonstrating its therapeutic efficacy against MI, especially via inhibiting the PI3K–AKT signaling pathway (Huang et al., 2022). The bioactive components and their protein targets were screened from *Salvia miltiorrhiza* Bunge using the TCMSP database. MI targets were obtained from the OMIM and GeneCards databases. GO and KEGG pathway enrichment analyses were performed to analyze the intersection of drug targets and disease targets using the Metascape database. Molecular docking results between active components and hub genes prioritized tanshinone IIA binding with vascular endothelial growth factor A (VEGFA). The experimental validation revealed that *Salvia miltiorrhiza* Bunge treats MI by promoting VEGF signaling pathway expression (Huang et al., 2023). The components of blood-entering Danshen Yin (DSY) were analyzed through UHPLC-Q-TOF-MS/MS. The SMILES structures of these components were retrieved via PubChem and input into the SwissTargetPrediction database for predicting potential targets. The DAVID database was utilized for KEGG pathway and GO enrichment analyses. Finally, integrating experimental findings, DSY may prevent myocardial fibrosis *in vivo* following MI by modulating transforming growth factor-β (TGF-β)-mediated PI3K–AKT, MAPK, and Smad signaling pathways (Gao X. et al., 2025). The active components of Ginseng and their targets were identified via the TCMSP database. The targets of MI and pyroptosis were collected from the GeneCards database. PPI, GO, and KEGG analyses of the intersection of drug targets and disease targets were carried out using STRING, Cytoscape, and DAVID databases. The molecular docking results between the active components and hub genes were obtained from AutoDock Vina and visualized using PyMOL software. Combined with experimental validation, the findings revealed that ginsenoside Rh2 reduced MI-induced cardiomyocyte pyroptosis via downregulating NOD-like receptor thermal protein domain-associated protein 3 (NLRP3), ASC, caspase-1, gasdermin-D (GSDMD-N), interleukin-18 (IL-18), and interleukin-1β (1L-1β) *in vivo* and *in vitro* (Li et al., 2025).

### Myocardial ischemia–reperfusion injury

Myocardial ischemia–reperfusion injury (MI/RI) is a pathological state caused by an initial low supply of blood to a specific area (ischemia), followed by the restoration of perfusion and re-oxygenation (reperfusion). The metabolites from the compounds of all ingredients in QishenYiqi (QSYQ) dripping pills were obtained from TCM and TCMIO. ChEMBL and TCMIO were used to identify the metabolite targets. An R-studio network was constructed to build a metabolite–target–pathway network. Molecular docking was performed between hub genes and the components of QSYQ. Finally, QSYQ was experimentally proven to alleviate MI/RI by regulating autophagy-related proteins and the PI3K–AKT–mammalian target of the rapamycin (mTOR) signaling pathway. QSYQ significantly suppressed pyroptosis via inhibiting the activation and assembly of NLRP3 inflammasome (Li et al., 2022). The components and their protein targets were screened from cinnamon and predicted using the TCMSP database and HyperAttentionDTI. A PPI network was constructed using the STRING database. For molecular docking, the homology modeling method was used to generate structures for proteins lacking unavailable structures, whereas AlphaFold2 predicted their 3D conformations. Blind docking was performed using QuickVina-W, and AutoDockTools defined the global docking box. Finally, experimental validation in a zebrafish model demonstrated that taxifolin exhibited potential protective effects against MI/RI via regulating PTGS2 (Xue et al., 2023). The components were screened from Shuxin decoction (SXT) via the TCMSP database. The diabetes-related targets or MI/RI-related targets were searched via the DisGeNET, GeneCards, DrugBank, OMIM, and PharmGKB databases. The key subnetworks and therapeutic targets were identified by the MCODE plugin in Cytoscape, and the CytoNCA plugin was further used to screen for core genes. KEGG and GO analyses were carried out through the DAVID database. Finally, combined with experimental validation, it was shown that SXT could significantly improve cardiac function in diabetic MI/RI by downregulating apoptosis-related proteins such as Bcl-2-associated X protein (Bax) and cleaved caspase-3 and upregulating Bcl-2 (Yang et al., 2023). Salvianolic acid B (Sal-B) targets were identified through screening databases and SwissTargetPrediction. The targets related to MI/RI were obtained by screening DisGeNET. The STRING database was used to construct a PPI network among the intersection between the targets of Sal-B and MI/RI. The network topology, GO, and KEGG were analyzed using Cytoscape and DAVID databases. Experimental results demonstrated that Sal-B may increase SIRT1 activity, inhibit the phosphorylation of c-Jun N-terminal kinase (JNK) and p38 mitogen-activated protein kinase (p38), reduce reactive oxygen species (ROS) release, and inhibit apoptosis (by increasing the ratio of Bcl-2/Bax and inhibiting caspase-3 activation) through the SIRT1–MAPK pathway (Mao et al., 2024).

### Heart failure

Heart failure (HF) is a chronic, progressive medical condition characterized by the heart’s inability to pump sufficient blood to meet the body’s metabolic needs or to do so only at elevated filling pressures. UPLC-QE-Orbitrap MS metabolite profiling identified 40 ginsenosides from the processed *Panax ginseng* C.A. Mey*.* [sun-dried ginseng (DG), red ginseng (RG), and black ginseng (BG)]. SwissTargetPrediction predicted potential ginseng targets, whereas OMIM, DisGeNET, and DrugBank provided HF targets. PPI network analysis identified the top hub genes using STRING and NetworkAnalyzer (BC, CC, and DC). Metascape GO/KEGG enrichment highlighted key pathways involved in treating HF. Molecular docking revealed that ginsenosides had the strongest binding force with mTOR in the treatment of HF (Dai et al., 2023). A total of 15 active components were identified from *Astragalus membranaceus* (HQ) using TCMSP. NPASS/PubChem were used to predict HQ targets. The overlapping downregulated and upregulated genes and their function between HQ targets and HF differentially expressed genes (DEGs) were yielded through GEO analysis. KEGG Mapper was exploited to perform signaling pathway enrichment annotation. PPI network analysis using STRING and CytoHubba’s MCC method in Cytoscape identified the top hub genes. ESR1 bound all key HQ components, as confirmed by AutoDockTools for molecular docking. HQ alleviated HF via ESR1-mediated pathways, inflammation, apoptosis, and vascular homeostasis (Chen et al., 2024). The components of ginseng and its protein targets were obtained and predicted through HERB, iTCM, and Comparative Toxicogenomics Database (CTD). The disease targets related to “HF” were retrieved from GeneCards, OMIM, DisGeNET, TTD, HPO, and MalaCards databases. The potential targets were collected from the intersection between drugs and disease targets and DEGs from the GEO database for enrichment analysis and PPI network. The hub genes were screened using Cytoscape. GO and KEGG enrichment analyses were performed on the hub genes using the clusterProfiler (version 4.10.1) package. Finally, the key components ginsenoside Rg1 and ginsenoside Rb3 were identified as the potential components in ginseng binding with fibronectin 1 (FN1) and protein kinase AMP-activated catalytic subunit alpha 2 (PRKAA2) involved in the PI3K–AKT and AMPK pathways through AutoDock Vina and *in vitro* and *in vivo* experiments (Gao K. et al., 2025). Active components of tonifying kidney and activating blood (KTBA) and its protein targets were screened and predicted from the TCMSP, SwissTargetPrediction, and PharmMapper databases. Chronic HF targets were extracted from GeneCards and DisGeNET databases. PPI network analysis of shared targets was performed using the STRING database and visualized in Cytoscape. The hub genes were identified via CytoNCA. GO and KEGG enrichment analyses via Metascape highlighted the key pathways. Molecular docking predicted that tanshinone IIA binds I-κBα/NF-κB pathway targets, suggesting that KTBA modulated chronic HF through multi-component interactions with inflammation/fibrosis-associated targets such as p38MAPK/NF-κB axis and epithelial barrier proteins including aquaporin-4 (AQP4)/zonula occludens-1 (ZO-1)/occludin (Xu et al., 2025).

### Drug-induced cardiotoxicity

Drug-induced cardiotoxicity (DIC) refers to the adverse cardiac effects caused by treatment with drugs, which may manifest as functional abnormalities. The active ginseng components and the corresponding targets were extracted from the TCMSP, HERB, and, ETCM databases. The DIC targets were identified as the overlapping targets of the DEGs from the GEO database. The core active components were screened using NetworkAnalyzer. PPI analysis was carried out through the STRING database and visualized in Cytoscape. The hub genes were identified through CytoNCA. GO and KEGG enrichment analyses identified the significant pathways using the “clusterProfiler” package, which were visualized in SangerBox. Molecular docking demonstrated that the core components bind with the hub genes, which was confirmed by cellular thermal shift assay (CETSA) and molecular dynamics (MD). Overall, the study revealed that kaempferol binds with STAT1 to protect against DIC (Xie et al., 2024). The DIC targets were identified through CTD and GeneCards. GO and KEGG enrichment analyses via the DAVID database elucidated the critical pathway. A PPI network was constructed using the STRING database and analyzed in Cytoscape to extract the hub genes. The animal experiments demonstrated that Danshensu (DSS), an active ingredient in *Salvia miltiorrhiza*, conferred cardioprotection by regulating the Kelch-like ECH-associated protein 1 (Keap1)/nuclear factor (erythroid-derived 2)-like 2 (Nrf2)/NAD(P)H quinone oxidoreductase 1 (NQO1) pathway, exerting synergistic antioxidant, anti-inflammatory, and anti-apoptotic effects (Qi et al., 2022). The Danshen injection (DSI)-associated targets were obtained from the TCMSP, HERB, ETCM, TCMID, and iTCM databases. The shared targets were subjected to PPI analysis through the STRING database, and the hub genes were analyzed in Cytoscape. GO and KEGG enrichment analyses identified the enriched pathways via the DAVID database, and the results were visualized in SangerBox. The transcriptome analysis validated the hub genes. Molecular docking was generated using the online software CB-Dock2 and demonstrated the high affinity of active compounds binding to the related targets. Overall, the study suggested that DSI conferred cardioprotection against AIC through inhibition of carbonic anhydrase 12 (CA12), nitric oxide synthase 3 (NOS3), and DNA polymerase eta (POLH), coupled with modulation of calcium signaling pathways (Xie et al., 2024).

### Diabetic cardiomyopathy

Diabetic cardiomyopathy (DCM) is a distinct myocardial disorder caused by long-term diabetes, and it is characterized by structural and functional abnormalities. The active components of *Hedysarum multijugum* Maxim-Radix Salviae compound (Huangqi–Danshen compound, HDC), containing *Hedysarum multijugum* and *Salvia miltiorrhiza*, were screened using TCM Database@Taiwan, TCMSP, and TCMID. Potential targets were predicted via PharmMapper using compound structures. After standardization in UniProtKB, the HDC targets were identified. DCM-related genes were extracted from GeneCards and OMIM databases. PPI analysis was performed using STRING with the network visualized in Cytoscape. GO and KEGG enrichment analyses via DAVID identified the key signaling pathways. Rat experiments showed that HDC reduced the levels of fasting plasma glucose (FPG), hemoglobin A1c (HbA1c), and malondialdehyde (MDA) and increased the levels of SOD and glutathione peroxidase (GSH-Px). The immunohistochemistry results showed that HDC could regulate the protein expression of apoptosis-related signaling pathways (increased Bcl-2 and decreased Bax) in DCM. The findings suggested that HDC could reduce oxidative stress and delay myocardial hypertrophy by inhibiting the AMPK–mTOR pathway, thereby improving myocardial damage (Zhang et al., 2020).

## Conclusion and perspectives

**FIGURE 2:**
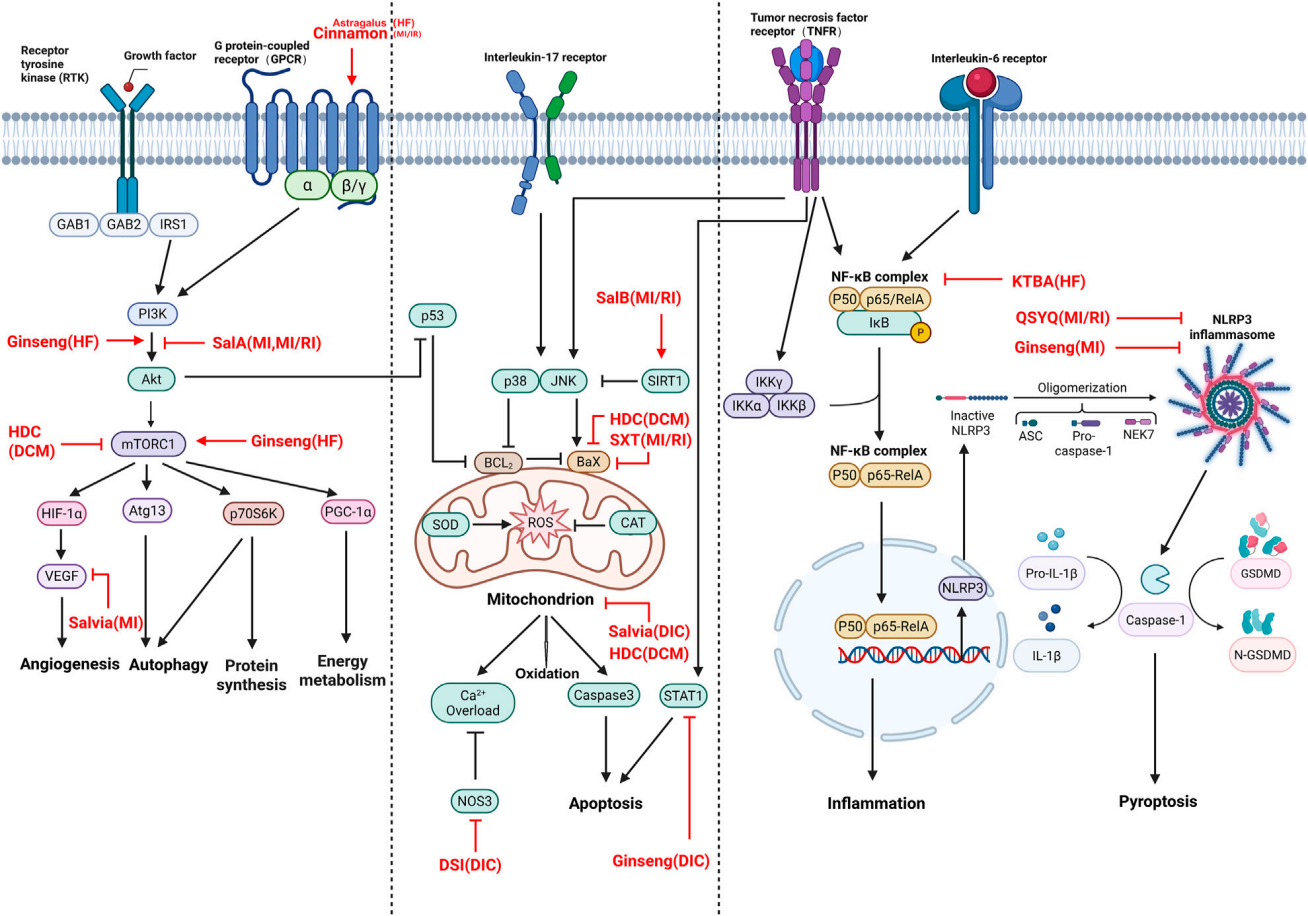
Schematic of shared signaling pathways treated by TCMs. MI, myocardial infarction; MI/RI, myocardial ischemia–reperfusion injury; HF, heart failure; DIC, drug-induced cardiotoxicity; DCM, diabetic cardiomyopathy. Sal-A, salvianolic acid A; Sal-B, salvianolic acid B; QSYQ, QishenYiqi dripping pill; SXT, Shuxin decoction; KTBA, tonifying kidney and activating blood; DSI, Danshen injection; HDC, Huangqi–Danshen compound; Salvia, Danshen; Astragalus, Huangqi; Ginseng, Renshen. This figure was created using BioRender, and its use has been authorized by the platform.

TCM and its main ingredients exert cardioprotective effects through key regulatory pathways against pathological conditions. Heart diseases include MI, MI/RI, HF, DIC, and DCM. The key herbs treat cardiac diseases primarily through the following mechanisms. First, it exerts anti-inflammatory and antioxidant effects by suppressing interleukin (IL) and TNF signaling pathways via NF-κB and decreasing the levels of ROS via balancing redox enzyme function. Second, it regulates the PI3K–AKT pathway and its pivotal downstream AKT–mTOR signaling pathway. PI3K–AKT–mTOR is widely involved in autophagy, cell growth, protein synthesis, energy metabolism, mitochondria biogenesis, and sensing and integration of upstream signaling pathways of growth factors and amino acids (Fan et al., 2025; Fan et al., 2022; Zhao T. et al., 2024). Third, it protects against pyroptosis through downregulating the assembly of NLRP3 inflammasome, inducing the release of caspase-1-dependent pro-inflammatory cytokines IL-1β and IL-18, along with gasdermin-D. Fourth, it directly influences apoptosis via upregulating Bcl-2 and downregulating p38, JNK, Bax, and caspase-3. Fifth, it promotes angiogenesis via enhancing VEGF signaling. Sixth, it maintains the calcium homeostasis via reinforcing NOS3 (Figure 2). The herbs share similar mechanisms through which they induce protective effects against MI and MI/RI, such as alleviating inflammation and pyroptosis, suppressing apoptosis, and inhibiting PI3K–AKT–mTOR to attenuate autophagy. In HF, TCM activates PI3K–AKT–mTOR to improve the energy metabolism, whereas it inhibits AMPK–mTOR to decrease hypertrophy via protein synthesis in DCM. Moreover, the herbs also protect against DIC and DCM both via anti-oxidation and anti-apoptosis.

Although NP has provided new insights into the treatment of cancer-induced cardiac injury, it has several limitations when used alone. (i) Much of the information in NP relies on computational predictions. Invalidated data concerning targets, pathway networks, and PPI inevitably lead to a high rate of false positives. For instance, molecular docking results can vary across different computational platforms. Therefore, experimental validation remains essential for confirming these predictions (Wu et al., 2022). (ii) NP prediction methods cannot accurately assess the efficacy and toxicological profile of TCM formulas. The chemical composition of some herbal ingredients changes during decoction processes. Consequently, the overall therapeutic effect of a herbal formula cannot be regarded as the simple combined effects of its individual compounds predicted *in silico*. For example, heating processes such as roasting and steaming can convert polar ginsenosides in raw ginseng into a variety of less polar ginsenosides (Li et al., 2018). (iii) Using only a few parameters such as OB and DL to represent the complex ADME process is an oversimplification. Many “active components” identified through NP may be present in very low concentrations in the raw herb or in biological fluids, potentially failing to reach effective therapeutic levels. Thus, the reliability of ADME-based screening for identifying truly active components remains questionable (Wu et al., 2022). These limitations also make translating the findings of NP to clinical application challenging.

Cancer-induced cardiac injury refers to cardiac dysfunction and wasting syndrome in cancer patients caused by the tumor itself, characterized by cardiac atrophy, cardiac fibrosis, and contractile dysfunction, with a dramatic impact on a patient’s quality of life and survival. Its pathogenesis primarily involves inflammatory responses, oxidative stress, protein degradation, and abnormal metabolism. TCM possesses anti-inflammatory and antioxidant effects and activates the AKT–mTOR pathway, especially attributing to protein synthesis and normal metabolism. These suggest that it may help improve cardiac injury. However, its effects on cancer-induced cardiac injury specifically have not yet been reported. Therefore, this represents a promising area for in-depth exploration. In addition, the approach to study the detailed mechanisms is also necessary. Hence, NP may be a useful and prospective method to investigate the new mechanisms. In therapy, understanding the specific mechanisms of the TCM and its active monomers holds important clinical implications. In future studies, we plan to use NP to identify potential therapeutic targets of key herbs, including *Astragalus membranaceus* (Huangqi), *Salvia miltiorrhiza* (Danshen), and Ginseng Radix (Renshen), for cancer-induced cardiac injury and validate these findings through *in vitro* and *in vivo* experiments.

## References

[B1] Amberger J. S. Bocchini C. A. Schiettecatte F. Scott A. F. Hamosh A. (2015). OMIM.org: Online mendelian inheritance in man (OMIM®), an online catalog of human genes and genetic disorders. Nucleic Acids Res. 43, D789–D798. 10.1093/nar/gku1205 25428349 PMC4383985

[B2] Arjmand B. Razzaghi M. Rezaei Tavirani M. Rostami-Nejad M. Rezaei Tavirani M. Vafaee R. (2022). Introducing critical common dysregulated proteins in esophageal, gastric, and intestinal cancers. Gastroenterol. Hepatol. Bed Bench. 15, 87–92. 35611247 PMC9123629

[B3] Barbarino J. M. Whirl-Carrillo M. Altman R. B. Klein T. E. (2018). PharmGKB: a worldwide resource for pharmacogenomic information. Wiley Interdiscip. Rev. Syst. Biol. Med. 10, e1417. 10.1002/wsbm.1417 29474005 PMC6002921

[B4] Barrett T. Wilhite S. E. Ledoux P. Evangelista C. Kim I. F. Tomashevsky M. (2013). NCBI GEO: archive for functional genomics data sets--update. Nucleic Acids Res. 41, D991–D995. 10.1093/nar/gks1193 23193258 PMC3531084

[B5] Berman H. M. Westbrook J. Feng Z. Gilliland G. Bhat T. N. Weissig H. (2000). The protein data bank. Nucleic Acids Res. 28, 235–242. 10.1093/nar/28.1.235 10592235 PMC102472

[B6] Bisht A. Tewari D. Kumar S. Chandra S. (2024). Network pharmacology, molecular docking, and molecular dynamics simulation to elucidate the mechanism of anti-aging action of Tinospora cordifolia. Mol. Divers 28, 1743–1763. 10.1007/s11030-023-10684-w 37439907

[B7] Burley S. K. Berman H. M. Kleywegt G. J. Markley J. L. Nakamura H. Velankar S. (2017). Protein data bank (PDB): the single global macromolecular structure archive. Methods Mol. Biol. 1607, 627–641. 10.1007/978-1-4939-7000-1_26 28573592 PMC5823500

[B8] Chen Q. Wang J. Sun L. Ba B. Shen D. (2024). Mechanism of Astragalus membranaceus (huangqi, HQ) for treatment of heart failure based on network pharmacology and molecular docking. J. Cell Mol. Med. 28, e18331. 10.1111/jcmm.18331 38780500 PMC11114218

[B9] Dai T. Gong J. Liu S. (2023). Prediction of molecular mechanism of processed ginseng in the treatment of heart failure based on network pharmacology and molecular docking technology. Med. Baltim. 102, e36576. 10.1097/md.0000000000036576 38065884 PMC10713105

[B10] Daina A. Michielin O. Zoete V. (2019). SwissTargetPrediction: updated data and new features for efficient prediction of protein targets of small molecules. Nucleic Acids Res. 47, W357–w364. 10.1093/nar/gkz382 31106366 PMC6602486

[B11] Fan J. Yuan Z. Burley S. K. Libutti S. K. Zheng X. F. S. (2022). Amino acids control blood glucose levels through mTOR signaling. Eur. J. Cell Biol. 101, 151240. 10.1016/j.ejcb.2022.151240 35623230 PMC10035058

[B12] Fan Y. Li Y. Fu X. Peng J. Chen Y. Chen T. (2023). Identification of potential ferroptosis key genes and immune infiltration in rheumatoid arthritis by integrated bioinformatics analysis. Heliyon 9, e21167. 10.1016/j.heliyon.2023.e21167 37920499 PMC10618794

[B13] Fan J. Khanzada Z. Xu Y. (2025). Mechanisms underlying muscle-related diseases and aging: insights into pathophysiology and therapeutic strategies. Muscles 4 (3), 26. 10.3390/muscles4030026 40843913 PMC12371960

[B14] Friboulet A. Thomas D. (2005). Systems Biology-an interdisciplinary approach. Biosens. Bioelectron. 20, 2404–2407. 10.1016/j.bios.2004.11.014 15854815

[B15] Gallo K. Goede A. Preissner R. Gohlke B. O. (2022). SuperPred 3.0: drug classification and target prediction-a machine learning approach. Nucleic Acids Res. 50, W726–w731. 10.1093/nar/gkac297 35524552 PMC9252837

[B16] Gao K. Xu D. Mu F. Zhao M. Zhang W. Tao X. (2025). Systems pharmacology to explore the potential mechanism of ginseng against heart failure. Rejuvenation Res. 28, 54–66. 10.1089/rej.2024.0051 39504983

[B17] Gao X. Ni C. Song Y. Xie X. Zhang S. Chen Y. (2025). Dan-shen yin attenuates myocardial fibrosis after myocardial infarction in rats: molecular mechanism insights by integrated transcriptomics and network pharmacology analysis and experimental validation. J. Ethnopharmacol. 338, 119070. 10.1016/j.jep.2024.119070 39522849

[B18] Gfeller D. Grosdidier A. Wirth M. Daina A. Michielin O. Zoete V. (2014). SwissTargetPrediction: a web server for target prediction of bioactive small molecules. Nucleic Acids Res. 42, W32–W38. 10.1093/nar/gku293 24792161 PMC4086140

[B19] Gong D. Yuan T. Wang R. Sun S. Dawuti A. Wang S. (2023). Network pharmacology approach and experimental verification of dan-shen decoction in the treatment of ischemic heart disease. Pharm. Biol. 61, 69–79. 10.1080/13880209.2022.2152059 36546685 PMC9793910

[B20] Hao P. Jiang F. Cheng J. Ma L. Zhang Y. Zhao Y. (2017). Traditional Chinese medicine for cardiovascular disease: evidence and potential mechanisms. J. Am. Coll. Cardiol. 69, 2952–2966. 10.1016/j.jacc.2017.04.041 28619197

[B21] He Q. Liu C. Wang X. Rong K. Zhu M. Duan L. (2023). Exploring the mechanism of curcumin in the treatment of colon cancer based on network pharmacology and molecular docking. Front. Pharmacol. 14, 1102581. 10.3389/fphar.2023.1102581 36874006 PMC9975159

[B22] Hopkins A. L. (2008). Network pharmacology: the next paradigm in drug discovery. Nat. Chem. Biol. 4, 682–690. 10.1038/nchembio.118 18936753

[B23] Huang Q. Zhang C. Tang S. Wu X. Peng X. (2022). Network pharmacology analyses of the pharmacological targets and therapeutic mechanisms of salvianolic acid A in myocardial infarction. Evid. Based Complement. Altern. Med. 2022, 8954035. 10.1155/2022/8954035 36248430 PMC9556248

[B24] Huang X. Zhang M. Song Y. Sun B. Lin L. Song X. (2023). Integrated network pharmacology to investigate the mechanism of Salvia miltiorrhiza bunge in the treatment of myocardial infarction. J. Cell Mol. Med. 27, 3514–3525. 10.1111/jcmm.17932 37643320 PMC10660626

[B25] Kim S. Chen J. Cheng T. Gindulyte A. He J. He S. (2019). PubChem 2019 update: improved access to chemical data. Nucleic Acids Res. 47, D1102–d1109. 10.1093/nar/gky1033 30371825 PMC6324075

[B26] Lan Y. Luo F. K. Yu Y. Wang X. Y. Wang P. Q. Xiong X. J. (2024). Coronary heart disease: innovative understanding from traditional Chinese medicine and treatment by classic formulas. Zhongguo Zhong Yao Za Zhi 49, 3684–3692. 10.19540/j.cnki.cjcmm.20240326.501 39041141

[B27] Li X. Yao F. Fan H. Li K. Sun L. Liu Y. (2018). Intraconversion of polar ginsenosides, their transformation into less-polar ginsenosides, and ginsenoside acetylation in ginseng flowers upon baking and steaming. Molecules 23 (4), 759. 10.3390/molecules23040759 29587462 PMC6017459

[B28] Li M. Wang Y. Qi Z. Yuan Z. Lv S. Zheng Y. (2022). QishenYiqi dripping pill protects against myocardial ischemia/reperfusion injury *via* suppressing excessive autophagy and NLRP3 inflammasome based on network pharmacology and experimental pharmacology. Front. Pharmacol. 13, 981206. 10.3389/fphar.2022.981206 36164369 PMC9507923

[B29] Li B. Mou S. Zhang C. Zhu T. Hu X. Li M. (2025). Ginsenoside Rh2 ameliorates myocardial infarction by regulating cardiomyocyte pyroptosis based on network pharmacology, molecular docking, and experimental verification. Am. J. Chin. Med. 53, 475–499. 10.1142/s0192415x25500181 40099395

[B30] Liu X. Ouyang S. Yu B. Liu Y. Huang K. Gong J. (2010). PharmMapper server: a web server for potential drug target identification using pharmacophore mapping approach. Nucleic Acids Res. 38, W609–W614. 10.1093/nar/gkq300 20430828 PMC2896160

[B31] Liu Z. Guo F. Wang Y. Li C. Zhang X. Li H. (2016). BATMAN-TCM: a bioinformatics analysis tool for molecular mechanism of traditional Chinese medicine. Sci. Rep. 6, 21146. 10.1038/srep21146 26879404 PMC4754750

[B32] Liu M. Yuan G. Luo G. Guo X. Chen M. Yang H. (2022). Network pharmacology analysis and experimental verification strategies reveal the action mechanism of danshen decoction in treating ischemic cardiomyopathy. Evid. Based Complement. Altern. Med. 2022, 7578055. 10.1155/2022/7578055 35722148 PMC9205745

[B33] Liu X. Yu Y. Wu Y. Luo A. Yang M. Li T. (2023). A systematic pharmacology-based *in vivo* study to reveal the effective mechanism of yupingfeng in asthma treatment. Phytomedicine 114, 154783. 10.1016/j.phymed.2023.154783 37004399

[B34] Liu Z. Shang Q. Li H. Fang D. Li Z. Huang Y. (2023). Exploring the possible mechanism(s) underlying the nephroprotective effect of zhenwu decoction in diabetic kidney disease: an integrated analysis. Phytomedicine 119, 154988. 10.1016/j.phymed.2023.154988 37523837

[B35] Mahmood S. S. Levy D. Vasan R. S. Wang T. J. (2014). The framingham heart study and the epidemiology of cardiovascular disease: a historical perspective. Lancet 383, 999–1008. 10.1016/s0140-6736(13)61752-3 24084292 PMC4159698

[B36] Mao Q. Shao C. Zhou H. Yu L. Bao Y. Zhao Y. (2024). Exploring the mechanism of salvianolic acid B against myocardial ischemia-reperfusion injury based on network pharmacology. Pharm. (Basel). 17 (3), 309. 10.3390/ph17030309 38543095 PMC10974641

[B37] Otasek D. Morris J. H. Bouças J. Pico A. R. Demchak B. (2019). Cytoscape automation: empowering workflow-based network analysis. Genome Biol. 20, 185. 10.1186/s13059-019-1758-4 31477170 PMC6717989

[B38] Piñero J. Ramírez-Anguita J. M. Saüch-Pitarch J. Ronzano F. Centeno E. Sanz F. (2020). The DisGeNET knowledge platform for disease genomics: 2019 update. Nucleic Acids Res. 48, D845–d855. 10.1093/nar/gkz1021 31680165 PMC7145631

[B39] Qi J. Y. Yang Y. K. Jiang C. Zhao Y. Wu Y. C. Han X. (2022). Exploring the mechanism of danshensu in the treatment of doxorubicin-induced cardiotoxicity based on network pharmacology and experimental evaluation. Front. Cardiovasc. Med. 9, 827975. 10.3389/fcvm.2022.827975 35295262 PMC8918531

[B40] Ren F. D. Liu Y. Z. Ding K. W. Chang L. L. Cao D. L. Liu S. (2024). Finite temperature string by K-means clustering sampling with order parameters as collective variables for molecular crystals: application to polymorphic transformation between β-CL-20 and ε-CL-20. Phys. Chem. Chem. Phys. 26, 3500–3515. 10.1039/d3cp05389j 38206084

[B41] Ru J. Li P. Wang J. Zhou W. Li B. Huang C. (2014). TCMSP: a database of systems pharmacology for drug discovery from herbal medicines. J. Cheminform. 6, 13. 10.1186/1758-2946-6-13 24735618 PMC4001360

[B42] Safran M. Dalah I. Alexander J. Rosen N. Iny Stein T. Shmoish M. (2010). GeneCards version 3: the human gene integrator. Database (Oxford) 2010, baq020. 10.1093/database/baq020 20689021 PMC2938269

[B43] Saito R. Smoot M. E. Ono K. Ruscheinski J. Wang P. L. Lotia S. (2012). A travel guide to cytoscape plugins. Nat. Methods 9, 1069–1076. 10.1038/nmeth.2212 23132118 PMC3649846

[B44] Shang L. Wang Y. Li J. Zhou F. Xiao K. Liu Y. (2023). Mechanism of sijunzi decoction in the treatment of colorectal cancer based on network pharmacology and experimental validation. J. Ethnopharmacol. 302, 115876. 10.1016/j.jep.2022.115876 36343798

[B45] Shannon P. Markiel A. Ozier O. Baliga N. S. Wang J. T. Ramage D. (2003). Cytoscape: a software environment for integrated models of biomolecular interaction networks. Genome Res. 13, 2498–2504. 10.1101/gr.1239303 14597658 PMC403769

[B46] Szklarczyk D. Gable A. L. Nastou K. C. Lyon D. Kirsch R. Pyysalo S. (2021). The STRING database in 2021: customizable protein-protein networks, and functional characterization of user-uploaded gene/measurement sets. Nucleic Acids Res. 49, D605–d612. 10.1093/nar/gkaa1074 33237311 PMC7779004

[B47] Trott O. Olson A. J. (2010). AutoDock vina: improving the speed and accuracy of docking with a new scoring function, efficient optimization, and multithreading. J. Comput. Chem. 31, 455–461. 10.1002/jcc.21334 19499576 PMC3041641

[B48] Wang Y. Wang Q. Li C. Lu L. Zhang Q. Zhu R. (2017). A review of Chinese herbal medicine for the treatment of chronic heart failure. Curr. Pharm. Des. 23, 5115–5124. 10.2174/1381612823666170925163427 28950815 PMC6340156

[B49] Wang T. Hou B. Qin H. Liang J. Shi M. Song Y. (2023). Qili qiangxin (QLQX) capsule as a multi-functional traditional Chinese medicine in treating chronic heart failure (CHF): a review of ingredients, molecular, cellular, and pharmacological mechanisms. Heliyon 9, e21950. 10.1016/j.heliyon.2023.e21950 38034785 PMC10682643

[B50] Wang A. Song Q. Li Y. Fang H. Ma X. Li Y. (2024). Effect of traditional Chinese medicine on metabolism disturbance in ischemic heart diseases. J. Ethnopharmacol. 329, 118143. 10.1016/j.jep.2024.118143 38583735

[B51] Wishart D. S. (2008). DrugBank and its relevance to pharmacogenomics. Pharmacogenomics 9, 1155–1162. 10.2217/14622416.9.8.1155 18681788

[B52] World health organization (2023). World health statistics 2023: monitoring health for the SDGs, sustainable development goals. World Health Organization. Available online at: https://www.who.int/publications/iitem/9789240074323.

[B53] Wu J. Zhang F. Li Z. Jin W. Shi Y. (2022). Integration strategy of network pharmacology in traditional Chinese medicine: a narrative review. J. Tradit. Chin. Med. 42 (3), 479–486. 10.19852/j.cnki.jtcm.20220408.003 35610020 PMC9924699

[B54] Xie L. Liu H. Zhang K. Pan Y. Chen M. Xue X. (2024). Exploring the molecular mechanism of ginseng against anthracycline-induced cardiotoxicity based on network pharmacology, molecular docking and molecular dynamics simulation. Hereditas 161, 31. 10.1186/s41065-024-00334-y 39243097 PMC11378563

[B55] Xu R. Bi Y. Ju Y. Yin W. Zhao S. Zhang Y. (2025). Uncovering the molecular mechanisms of tonifying kidney and activating blood decoction against myocardial fibrosis using network pharmacology and experimental validation. Sci. Rep. 15, 18912. 10.1038/s41598-025-01276-9 40442166 PMC12122709

[B56] Xue T. Xue Y. Fang Y. Lu C. Fu Y. Lai Z. (2023). Exploring myocardial ischemia-reperfusion injury mechanism of cinnamon by network pharmacology, molecular docking, and experiment validation. Comput. Math. Methods Med. 2023, 1066057. 10.1155/2023/1066057 36873789 PMC9981296

[B57] Yang L. Jian Y. Zhang Z. Y. Qi B. W. Li Y. B. Long P. (2023). Network-pharmacology-based research on protective effects and underlying mechanism of shuxin decoction against myocardial ischemia/reperfusion injury with diabetes. World J. Diabetes 14, 1057–1076. 10.4239/wjd.v14.i7.1057 37547579 PMC10401449

[B58] Zeng X. Zhang P. He W. Qin C. Chen S. Tao L. (2018). NPASS: natural product activity and species source database for natural product research, discovery and tool development. Nucleic Acids Res. 46, D1217–d1222. 10.1093/nar/gkx1026 29106619 PMC5753227

[B59] Zhang S. Yuan Z. Wu H. Li W. Li L. Huang H. (2020). Network pharmacology-based strategy reveals the effects of Hedysarum multijugum maxim.-radix salviae compound on oxidative capacity and cardiomyocyte apoptosis in rats with diabetic cardiomyopathy. Biomed. Res. Int. 2020, 8260703. 10.1155/2020/8260703 33134388 PMC7591987

[B60] Zhang P. Zhang D. Zhou W. Wang L. Wang B. Zhang T. (2023). Network pharmacology: towards the artificial intelligence-based precision traditional Chinese medicine. Brief. Bioinform. 25, bbad518. 10.1093/bib/bbad518 38197310 PMC10777171

[B61] Zhao C. Sahni S. (2019). String correction using the damerau-levenshtein distance. BMC Bioinforma. 20 (Suppl. 11), 277. 10.1186/s12859-019-2819-0 31167641 PMC6551241

[B62] Zhao M. Feng L. Li W. (2024). Network pharmacology and experimental verification: sanqi-danshen treats coronary heart disease by inhibiting the PI3K/AKT signaling pathway. Drug Des. Devel. Ther. 18, 4529–4550. 10.2147/dddt.S480248 39399124 PMC11471080

[B63] Zhao T. Fan J. Abu-Zaid A. Burley S. K. Zheng X. F. S. (2024). Nuclear mTOR signaling orchestrates transcriptional programs underlying cellular growth and metabolism. Cells 13, 781. 10.3390/cells13090781 38727317 PMC11083943

[B64] Zhou Y. Zhang Y. Zhao D. Yu X. Shen X. Zhou Y. (2024). TTD: therapeutic target database describing target druggability information. Nucleic Acids Res. 52, D1465–d1477. 10.1093/nar/gkad751 37713619 PMC10767903

